# Supplementary material for: Targeted NGS-Based Analysis of Pneumocystis jirovecii Reveals Novel Genotypes
Source: J Fungi (Basel). 2022 Aug 17;8(8):863. doi: 10.3390/jof8080863 (PMC9409852; doi:10.3390/jof8080863)
Supplement: Supplementary file 1 [file jof-08-00863-s001.zip › SupplementaryFiles/Supplemental Table 1.pdf]

Supplemental Table 1

Sales Order	Reference	Manufacturing ID	Product	Purification	Sequence Name	Target	Unit Size	Bases	Sequence	Anhydrous Molecular Weight	nmoles/OD	ug/OD	Extinction Coefficient	GC Content	Tm (50mM NaCl) C	Modifications and Services	Final OD	nmoles	Conc	Volume	Buffer	Print Date	Well Position
17195044	285828293	355925386	Custom rhAmpSeq™ FWD Panel, 0.4 nmol		RHH.W4300B6A5BDF041			-1	N/A									nmoles	N/A	N/A	N/A		
17195044	285828294	355124441	rhAmpSeq™ Primer (2mL Rack - 1600µL)	Standard Desalting	RH.CF78E0180F584D9Z0Z.F	T551_03199	0.025	29	/rhSeq-f/TCA TAG CCC ACA TGA ATA ACA ATT rCCA GA/GT4/	19225.4	1.717917884	33.02771173	582100	37.93103448	58.28548646	rhSeq-f GT4 Standard Desalting	N/A	0.25µM in 1600µLIDTE Buffer pH 7.5	0.25µM	1600µL	IDTE Buffer pH 7.5	12/14/2021 13:43	
17195044	285828295	355124442	rhAmpSeq™ Primer (2mL Rack - 1600µL)	Standard Desalting	RH.A2C31F6013C24B7Z0Z.F	T551_03199	0.025	28	/rhSeq-f/GTT CAA TGC TGA CAC TGT TTA TCrG CCA T/GT4/	18932.2	1.820498817	34.46603495	549300	42.85714286	59.2205463	rhSeq-f GT4 Standard Desalting	N/A	0.25µM in 1600µLIDTE Buffer pH 7.5	0.25µM	1600µL	IDTE Buffer pH 7.5	12/14/2021 13:43	
17195044	285828296	355124443	rhAmpSeq™ Primer (2mL Rack - 1600µL)	Standard Desalting	RH.1B63D59F4F974A1Z0Z.F	?T551_02857	0.025	31	/rhSeq-f/GGT TTC TGA GAA AGA TTT AGC CAT ATrG TAA C/GT1/	19999.9	1.659200265	33.18386926	602700	35.48387097	55.73220926	rhSeq-f GT1 Standard Desalting	N/A	0.25µM in 1600µLIDTE Buffer pH 7.5	0.25µM	1600µL	IDTE Buffer pH 7.5	12/14/2021 13:43	
17195044	285828297	355124444	rhAmpSeq™ Primer (2mL Rack - 1600µL)	Standard Desalting	RH.FD38EF6B8AEA421Z0Z.F	T551_00303	0.025	25	/rhSeq-f/GAT CCA CTG TCC AGA CAA TTrG CAT C/GT1/	18012.6	1.895734597	34.14714502	527500	48	58.49605875	rhSeq-f GT1 Standard Desalting	N/A	0.25µM in 1600µLIDTE Buffer pH 7.5	0.25µM	1600µL	IDTE Buffer pH 7.5	12/14/2021 13:43	
17195044	285828298	355124445	rhAmpSeq™ Primer (2mL Rack - 1600µL)	Standard Desalting	RH.103374AC8028435Z0Z.F	T551_02533	0.025	26	/rhSeq-f/ATA GGA GTG GTT TCC GTC TTT rCTT TC/GT3/	18391.8	1.889644747	34.75400983	529200	42.30769231	56.54352354	rhSeq-f GT3 Standard Desalting	N/A	0.25µM in 1600µLIDTE Buffer pH 7.5	0.25µM	1600µL	IDTE Buffer pH 7.5	12/14/2021 13:43	
17195044	285828299	355124446	rhAmpSeq™ Primer (2mL Rack - 1600µL)	Standard Desalting	RH.E0CE477C4B19464Z0Z.F	T551_00896	0.025	28	/rhSeq-f/GAG AAA ATG ATA GAC TTA GGG CTrC AAA A/GT4/	19105.4	1.698081168	32.44244524	588900	35.71428571	54.70990745	rhSeq-f GT4 Standard Desalting	N/A	0.25µM in 1600µLIDTE Buffer pH 7.5	0.25µM	1600µL	IDTE Buffer pH 7.5	12/14/2021 13:43	
17195044	285828300	355124447	rhAmpSeq™ Primer (2mL Rack - 1600µL)	Standard Desalting	RH.316DF1391E794B9Z0Z.F	T551_01756	0.025	26	/rhSeq-f/CAG AAG ATG AAT TGC CAC CAA rACA TT/GT4/	18358.9	1.813236627	33.28896827	551500	38.46153846	57.30854281	rhSeq-f GT4 Standard Desalting	N/A	0.25µM in 1600µLIDTE Buffer pH 7.5	0.25µM	1600µL	IDTE Buffer pH 7.5	12/14/2021 13:43	
17195044	285828301	355124448	rhAmpSeq™ Primer (2mL Rack - 1600µL)	Standard Desalting	RH.E45C51A8E1AB462Z0Z.F	T551_01755	0.025	28	/rhSeq-f/CAT CTG CAG GTA AAG ACA AGA AArC CAG A/GT1/	19059.3	1.715854496	32.70304221	582800	42.85714286	59.5389217	rhSeq-f GT1 Standard Desalting	N/A	0.25µM in 1600µLIDTE Buffer pH 7.5	0.25µM	1600µL	IDTE Buffer pH 7.5	12/14/2021 13:43	
17195044	285828302	355124449	rhAmpSeq™ Primer (2mL Rack - 1600µL)	Standard Desalting	RH.AD8B9A3AFA344A3Z0Z.F	?T551_01830	0.025	23	/rhSeq-f/CCC GAA TTT CCG TCC AAT rCAG AC/GT2/	17346.2	1.984126984	34.41701786	504000	52.17391304	57.57764664	rhSeq-f GT2 Standard Desalting	N/A	0.25µM in 1600µLIDTE Buffer pH 7.5	0.25µM	1600µL	IDTE Buffer pH 7.5	12/14/2021 13:43	
17195044	285828303	355124450	rhAmpSeq™ Primer (2mL Rack - 1600µL)	Standard Desalting	RH.47196AD4997D471Z0Z.F	?T551_03041 ?T551_02080	0.025	27	/rhSeq-f/TGA CAC ACA AAG GCC ATT TAT TrCT GTC /GT1/	18645	1.823154057	33.99277302	548500	40.74074074	57.55322918	rhSeq-f GT1 Standard Desalting	N/A	0.25µM in 1600µLIDTE Buffer pH 7.5	0.25µM	1600µL	IDTE Buffer pH 7.5	12/14/2021 13:43	
17195044	285828304	355124451	rhAmpSeq™ Primer (2mL Rack - 1600µL)	Standard Desalting	RH.AEC03A6FD9A64E0Z0Z.F	T551_03158	0.025	27	/rhSeq-f/CCG AAT TGG AAC TCA CTT TAC TrCG AAG /GT3/	18686	1.821161901	34.03031688	549100	44.44444444	56.68720574	rhSeq-f GT3 Standard Desalting	N/A	0.25µM in 1600µLIDTE Buffer pH 7.5	0.25µM	1600µL	IDTE Buffer pH 7.5	12/14/2021 13:43	
17195044	285828305	355124452	rhAmpSeq™ Primer (2mL Rack - 1600µL)	Standard Desalting	RH.E8A3CF8496644ACZ0Z.F	T551_02309	0.025	29	/rhSeq-f/TCT GGA GTA AAT CGA ACT TGA TTT rGCT TC/GT4/	19300.4	1.769285209	34.14797948	565200	37.93103448	57.73649278	rhSeq-f GT4 Standard Desalting	N/A	0.25µM in 1600µLIDTE Buffer pH 7.5	0.25µM	1600µL	IDTE Buffer pH 7.5	12/14/2021 13:43	
17195044	285828306	355124453	rhAmpSeq™ Primer (2mL Rack - 1600µL)	Standard Desalting	RH.8DF9507B0841432Z0Z.F	mito NAD6	0.025	32	/rhSeq-f/CAA TTC TGG TTG TAT CTT CAA GAA ATC rCTG TA/GT2/	20206	1.668335002	33.71044378	599400	34.375	56.86876111	rhSeq-f GT2 Standard Desalting	N/A	0.25µM in 1600µLIDTE Buffer pH 7.5	0.25µM	1600µL	IDTE Buffer pH 7.5	12/14/2021 13:43	
17195044	285828307	355124454	rhAmpSeq™ Primer (2mL Rack - 1600µL)	Standard Desalting	RH.3E370038B0CB4B3Z0Z.F	mito NAD1	0.025	30	/rhSeq-f/GCT TTA TTA GGT TCC TTG TGA AGT ArCT GCT /GT3/	19651.6	1.757469244	34.53715993	569000	40	58.94277635	rhSeq-f GT3 Standard Desalting	N/A	0.25µM in 1600µLIDTE Buffer pH 7.5	0.25µM	1600µL	IDTE Buffer pH 7.5	12/14/2021 13:43	
17195044	285828308	355124455	rhAmpSeq™ Primer (2mL Rack - 1600µL)	Standard Desalting	RH.DDFCD6AD33494FBZ0Z.F	cytochrome c oxidase subunit 1	0.025	25	/rhSeq-f/TGA TTT CAC CAC CAG CTT TCrC ATG C/GT1/	17954.6	1.950458358	35.01963917	512700	48	59.60946329	rhSeq-f GT1 Standard Desalting	N/A	0.25µM in 1600µLIDTE Buffer pH 7.5	0.25µM	1600µL	IDTE Buffer pH 7.5	12/14/2021 13:43	
17195044	285828309	355124456	rhAmpSeq™ Primer (2mL Rack - 1600µL)	Standard Desalting	RH.FF72BC52B1E14BCZ0Z.F	atp6	0.025	28	/rhSeq-f/CTT CAC ATC TGG TCT TTA CTG TCrG CTT T/GT3/	18905.2	1.879699248	35.53600376	532000	42.85714286	58.39315957	rhSeq-f GT3 Standard Desalting	N/A	0.25µM in 1600µLIDTE Buffer pH 7.5	0.25µM	1600µL	IDTE Buffer pH 7.5	12/14/2021 13:43	
17195044	285828310	355124457	rhAmpSeq™ Primer (2mL Rack - 1600µL)	Standard Desalting	RH.E67274DA8AD04D9Z0Z.F	cytochrome c oxidase subunit 3	0.025	29	/rhSeq-f/CTA GCT TTT ACT CTT TTA CAG GGT rGTG GA/GT4/	19307.4	1.77999288	34.36707725	561800	41.37931034	57.8157016	rhSeq-f GT4 Standard Desalting	N/A	0.25µM in 1600µLIDTE Buffer pH 7.5	0.25µM	1600µL	IDTE Buffer pH 7.5	12/14/2021 13:43	
17195044	285828311	355124458	rhAmpSeq™ Primer (2mL Rack - 1600µL)	Standard Desalting	RH.EF9CF32F289649AZ0Z.F	atp9	0.025	26	/rhSeq-f/GGT TCA GGG TTA GCT ACA ATT rGGA TT/GT4/	18458.9	1.832844575	33.83226723	545600	42.30769231	57.50869948	rhSeq-f GT4 Standard Desalting	N/A	0.25µM in 1600µLIDTE Buffer pH 7.5	0.25µM	1600µL	IDTE Buffer pH 7.5	12/14/2021 13:43	
17195044	285828312	355124459	rhAmpSeq™ Primer (2mL Rack - 1600µL)	Standard Desalting	RH.99229D6013E14CBZ0Z.F	NADH dehydrogenase subunit 4L	0.025	30	/rhSeq-f/TCC ATG GCT TTA GAT GAT TTA GAA GrGA CAG /GT1/	19711.7	1.694053871	33.39271726	590300	40	58.75456205	rhSeq-f GT1 Standard Desalting	N/A	0.25µM in 1600µLIDTE Buffer pH 7.5	0.25µM	1600µL	IDTE Buffer pH 7.5	12/14/2021 13:43	
17195044	285828313	355124460	rhAmpSeq™ Primer (2mL Rack - 1600µL)	Standard Desalting	RH.795964E1E42344EZ0Z.F		0.025	27	/rhSeq-f/TCA CTC CTT TGG TCT ATA CTG TrCT GTG /GT1/	18625	1.883593897	35.08190431	530900	44.44444444	57.39996768	rhSeq-f GT1 Standard Desalting	N/A	0.25µM in 1600µLIDTE Buffer pH 7.5	0.25µM	1600µL	IDTE Buffer pH 7.5	12/14/2021 13:43	
17195044	285828314	355925384	Custom rhAmpSeq™ REV Panel, 0.4 nmol		RHH.W4300B6A5BDF041			-1	N/A									nmoles	N/A	N/A	N/A		
17195044	285828315	355124461	rhAmpSeq™ Primer (2mL Rack - 1600µL)	Standard Desalting	RH.CF78E0180F584D9Z0Z.R	T551_03199	0.025	28	/rhSeq-r/CGT GAT AAA GAA CTT GCA ACA TGrC ACA T/GT1/	19576.6	1.679261125	32.87428044	595500	39.28571429	58.3959983	rhSeq-r GT1 Standard Desalting	N/A	0.25µM in 1600µLIDTE Buffer pH 7.5	0.25µM	1600µL	IDTE Buffer pH 7.5	12/14/2021 13:43	
17195044	285828316	355124462	rhAmpSeq™ Primer (2mL Rack - 1600µL)	Standard Desalting	RH.A2C31F6013C24B7Z0Z.R	T551_03199	0.025	29	/rhSeq-r/GAG AGT ATT TGG CTC AAA AAG AAG rCGG AG/GT4/	20026.9	1.615508885	32.35363651	619000	44.82758621	59.0481418	rhSeq-r GT4 Standard Desalting	N/A	0.25µM in 1600µLIDTE Buffer pH 7.5	0.25µM	1600µL	IDTE Buffer pH 7.5	12/14/2021 13:43	
17195044	285828317	355124463	rhAmpSeq™ Primer (2mL Rack - 1600µL)	Standard Desalting	RH.1B63D59F4F974A1Z0Z.R	T551_03038	0.025	32	/rhSeq-r/TCT ACT CTA TTA ATG TAT TCT GAC CCA rCAA GG/GT4/	20696.3	1.610305958	33.32742512	621000	37.5	57.45960869	rhSeq-r GT4 Standard Desalting	N/A	0.25µM in 1600µLIDTE Buffer pH 7.5	0.25µM	1600µL	IDTE Buffer pH 7.5	12/14/2021 13:43	
17195044	285828318	355124464	rhAmpSeq™ Primer (2mL Rack - 1600µL)	Standard Desalting	RH.FD38EF6B8AEA421Z0Z.R	T551_00303	0.025	25	/rhSeq-r/GGT TAT GAC AAT TTC GCC GArC CGT T/GT2/	18626	1.815541031	33.81620733	550800	48	60.75896139	rhSeq-r GT2 Standard Desalting	N/A	0.25µM in 1600µLIDTE Buffer pH 7.5	0.25µM	1600µL	IDTE Buffer pH 7.5	12/14/2021 13:43	
17195044	285828319	355124465	rhAmpSeq™ Primer (2mL Rack - 1600µL)	Standard Desalting	RH.103374AC8028435Z0Z.R	T551_02533	0.025	26	/rhSeq-r/TGA CCA AGT CGC TGT TAT TGT rATA TG/GT2/	18969.2	1.758396343	33.3553719	568700	38.46153846	54.89064616	rhSeq-r GT2 Standard Desalting	N/A	0.25µM in 1600µLIDTE Buffer pH 7.5	0.25µM	1600µL	IDTE Buffer pH 7.5	12/14/2021 13:43	
17195044	285828320	355124466	rhAmpSeq™ Primer (2mL Rack - 1600µL)	Standard Desalting	RH.E0CE477C4B19464Z0Z.R	?T551_00896	0.025	26	/rhSeq-r/GAT GTT TGC TTG GCT AAG GTA rCAG AT/GT1/	19043.2	1.755617978	33.43267029	569600	42.30769231	57.46759574	rhSeq-r GT1 Standard Desalting	N/A	0.25µM in 1600µLIDTE Buffer pH 7.5	0.25µM	1600µL	IDTE Buffer pH 7.5	12/14/2021 13:43	
17195044	285828321	355124467	rhAmpSeq™ Primer (2mL Rack - 1600µL)	Standard Desalting	RH.316DF1391E794B9Z0Z.R	T551_01756	0.025	26	/rhSeq-r/TCC CAT CGT AAA AGG CGT AAT rCTT GA/GT1/	18941.2	1.747640685	33.10241699	572200	42.30769231	57.95112365	rhSeq-r GT1 Standard Desalting	N/A	0.25µM in 1600µLIDTE Buffer pH 7.5	0.25µM	1600µL	IDTE Buffer pH 7.5	12/14/2021 13:43	
17195044	285828322	355124468	rhAmpSeq™ Primer (2mL Rack - 1600µL)	Standard Desalting	RH.E45C51A8E1AB462Z0Z.R	T551_01755	0.025	29	/rhSeq-r/TGA ACA AAT GAA TGC GAC AAT AGT rCCC AA/GT2/	19889.8	1.625223468	32.32543962	615300	37.93103448	59.14967693	rhSeq-r GT2 Standard Desalting	N/A	0.25µM in 1600µLIDTE Buffer pH 7.5	0.25µM	1600µL	IDTE Buffer pH 7.5	12/14/2021 13:43	
17195044	285828323	355124469	rhAmpSeq™ Primer (2mL Rack - 1600µL)	Standard Desalting	RH.AD8B9A3AFA344A3Z0Z.R	T551_01830	0.025	24	/rhSeq-r/TCA AGG GAA TCG AAC CAC ArCC ATC /GT4/	18262.8	1.810938066	33.07273271	552200	50	60.0796593	rhSeq-r GT4 Standard Desalting	N/A	0.25µM in 1600µLIDTE Buffer pH 7.5	0.25µM	1600µL	IDTE Buffer pH 7.5	12/14/2021 13:43	
17195044	285828324	355124470	rhAmpSeq™ Primer (2mL Rack - 1600µL)	Standard Desalting	RH.47196AD4997D471Z0Z.R	T551_02650	0.025	28	/rhSeq-r/TTT GTT TGG TGC AAG CAT TAT TTrC AAG G/GT2/	19617.6	1.714089818	33.62635585	583400	35.71428571	56.90069548	rhSeq-r GT2 Standard Desalting	N/A	0.25µM in 1600µLIDTE Buffer pH 7.5	0.25µM	1600µL	IDTE Buffer pH 7.5	12/14/2021 13:43	
17195044	285828325	355124471	rhAmpSeq™ Primer (2mL Rack - 1600µL)	Standard Desalting	RH.AEC03A6FD9A64E0Z0Z.R	T551_03158	0.025	26	/rhSeq-r/CGT GAC CAG GAT ACG ATA TCT rCAT GG/GT3/	18998.2	1.745505324	33.16148193	572900	50	57.82730302	rhSeq-r GT3 Standard Desalting	N/A	0.25µM in 1600µLIDTE Buffer pH 7.5	0.25µM	1600µL	IDTE Buffer pH 7.5	12/14/2021 13:43	
17195044	285828326	355124472	rhAmpSeq™ Primer (2mL Rack - 1600µL)	Standard Desalting	RH.E8A3CF8496644ACZ0Z.R	T551_02309	0.025	30	/rhSeq-r/CCT TTA GAA TCT GCA ATA TAT CGT TrGG AAG /GT4/	20183	1.635322976	33.005722	611500	36.66666667	56.16303448	rhSeq-r GT4 Standard Desalting	N/A	0.25µM in 1600µLIDTE Buffer pH 7.5	0.25µM	1600µL	IDTE Buffer pH 7.5	12/14/2021 13:43	
17195044	285828327	354990802	rhAmpSeq™ Primer (2mL Rack - 1600µL)	Standard Desalting	RH.8DF9507B0841432Z0Z.R	mito NAD6	0.025	31	/rhSeq-r/GCT ATA GCT CCA ACA TAT ACA GTA ATrA TAT G/GT4/	20449.2	1.579778831	32.30521011	633000	32.25806452	53.18217056	rhSeq-r GT4 Standard Desalting	N/A	0.25µM in 1600µLIDTE Buffer pH 7.5	0.25µM	1600µL	IDTE Buffer pH 7.5	12/14/2021 13:43	
17195044	285828328	354990803	rhAmpSeq™ Primer (2mL Rack - 1600µL)	Standard Desalting	RH.3E370038B0CB4B3Z0Z.R	mito NAD1	0.025	30	/rhSeq-r/GGC AAA ACA AAC CAA ATA GCT TTT TrGT TCT /GT1/	20160	1.642845408	33.11978643	608700	33.33333333	57.36155069	rhSeq-r GT1 Standard Desalting	N/A	0.25µM in 1600µLIDTE Buffer pH 7.5	0.25µM	1600µL	IDTE Buffer pH 7.5	12/14/2021 13:43	
17195044	285828329	354990804	rhAmpSeq™ Primer (2mL Rack - 1600µL)	Standard Desalting	RH.DDFCD6AD33494FBZ0Z.R	atp8	0.025	26	/rhSeq-r/TGT CTA GGC AGA ACC CAT TTA rGCG AA/GT2/	18957.2	1.747946163	33.1361685	572100	46.15384615	60.79604718	rhSeq-r GT2 Standard Desalting	N/A	0.25µM in 1600µLIDTE Buffer pH 7.5	0.25µM	1600µL	IDTE Buffer pH 7.5	12/14/2021 13:43	
17195044	285828330	354990805	rhAmpSeq™ Primer (2mL Rack - 1600µL)	Standard Desalting	RH.FF72BC52B1E14BCZ0Z.R	atp6	0.025	29	/rhSeq-r/ACA AAG AAG GAG AAG AAT TTC AGA rCCA TG/GT1/	19987.9	1.59642401	31.90919061	626400	37.93103448	58.01863944	rhSeq-r GT1 Standard Desalting	N/A	0.25µM in 1600µLIDTE Buffer pH 7.5	0.25µM	1600µL	IDTE Buffer pH 7.5	12/14/2021 13:43	
17195044	285828331	354990806	rhAmpSeq™ Primer (2mL Rack - 1600µL)	Standard Desalting	RH.E67274DA8AD04D9Z0Z.R	cytochrome c oxidase subunit 3	0.025	29	/rhSeq-r/AGT CCT ACA GAT AAA AAG ATA GCA rCCC AC/GT1/	19843.8	1.629195178	32.32945585	613800	41.37931034	58.71021286	rhSeq-r GT1 Standard Desalting	N/A	0.25µM in 1600µLIDTE Buffer pH 7.5	0.25µM	1600µL	IDTE Buffer pH 7.5	12/14/2021 13:43	
17195044	285828332	354990807	rhAmpSeq™ Primer (2mL Rack - 1600µL)	Standard Desalting	RH.EF9CF32F289649AZ0Z.R	atp9	0.025	25	/rhSeq-r/GCT TCT GCT AGA GCA AAT CCrC AAG A/GT1/	18622	1.791151711	33.3548182	558300	48	58.5609773	rhSeq-r GT1 Standard Desalting	N/A	0.25µM in 1600µLIDTE Buffer pH 7.5	0.25µM	1600µL	IDTE Buffer pH 7.5	12/14/2021 13:43	
17195044	285828333	354990808	rhAmpSeq™ Primer (2mL Rack - 1600µL)	Standard Desalting	RH.99229D6013E14CBZ0Z.R	Nad5	0.025	29	/rhSeq-r/ACA CCG ATT TTA TGT CCT AAC AAA rCCT AC/GT4/	19727.7	1.682368775	33.18930013	594400	37.93103448	57.79180513	rhSeq-r GT4 Standard Desalting	N/A	0.25µM in 1600µLIDTE Buffer pH 7.5	0.25µM	1600µL	IDTE Buffer pH 7.5	12/14/2021 13:43	
17195044	285828334	354990809	rhAmpSeq™ Primer (2mL Rack - 1600µL)	Standard Desalting	RH.795964E1E42344EZ0Z.R	Nad4	0.025	26	/rhSeq-r/GCC AAA GCC ATA TGT CCA ATA rGAA GA/GT4/	18944.2	1.711449598	32.42207257	584300	42.30769231	57.72449592	rhSeq-r GT4 Standard Desalting	N/A	0.25µM in 1600µLIDTE Buffer pH 7.5	0.25µM	1600µL	IDTE Buffer pH 7.5	12/14/2021 13:43	
17195044	285828336	354978057	rhAmpSeq™ Index 1 (i5) Primer, 6 nmol	Standard Desalting	i5_IDT-1		0.025	57	AAT GAT ACG GCG ACC ACC GAG ATC TAC ACA TAT GCG CAC ACT CTT TCC CTA CAC GAC	17355.3	1.842299189	31.97364959	542800	50.87719298	70.15320933	Standard Desalting	N/A	6nmol	N/A	N/A	N/A	12/14/2021 13:43	
17195044	285828337	354978058	rhAmpSeq™ Index 2 (i5) Primer, 6 nmol	Standard Desalting	i5_IDT-2		0.025	57	AAT GAT ACG GCG ACC ACC GAG ATC TAC ACT GGT ACA GAC ACT CTT TCC CTA CAC GAC	17395.3	1.828487841	31.80713293	546900	50.87719298	69.69013058	Standard Desalting	N/A	6nmol	N/A	N/A	N/A	12/14/2021 13:43	
17195044	285828338	354978059	rhAmpSeq™ Index 1 (i7) Primer, 6 nmol	Standard Desalting	i7_IDT-1		0.025	53	CAA GCA GAA GAC GGC ATA CGA GAT ACG ATC AGG TGA CTG GAG TTC AGA CGT GT	16488.7	1.857010214	30.61973816	538500	50.94339623	69.47003643	Standard Desalting	N/A	6nmol	N/A	N/A	N/A	12/14/2021 13:43	
17195044	285828339	354978060	rhAmpSeq™ Index 2 (i7) Primer, 6 nmol	Standard Desalting	i7_IDT-2		0.025	53	CAA GCA GAA GAC GGC ATA CGA GAT TCG AGA GTG TGA CTG GAG TTC AGA CGT GT	16519.7	1.862544235	30.76874651	536900	50.94339623	69.56324728	Standard Desalting	N/A	6nmol	N/A	N/A	N/A	12/14/2021 13:43	
17195044	285828340	354978061	rhAmpSeq™ Index 3 (i7) Primer, 6 nmol	Standard Desalting	i7_IDT-3		0.025	53	CAA GCA GAA GAC GGC ATA CGA GAT CTA GCT CAG TGA CTG GAG TTC AGA CGT GT	16439.7	1.887861053	31.03585426	529700	50.94339623	69.39832188	Standard Desalting	N/A	6nmol	N/A	N/A	N/A	12/14/2021 13:43	
17195044	285828341	354978062	rhAmpSeq™ Index 4 (i7) Primer, 6 nmol	Standard Desalting	i7_IDT-4		0.025	53	CAA GCA GAA GAC GGC ATA CGA GAT ATC GTC TCG TGA CTG GAG TTC AGA CGT GT	16430.7	1.896094046	31.15411263	527400	50.94339623	69.34481251	Standard Desalting	N/A	6nmol	N/A	N/A	N/A	12/14/2021 13:43	
17195044	285828342	354978063	rhAmpSeq™ Index 5 (i7) Primer, 6 nmol	Standard Desalting	i7_IDT-5		0.025	53	CAA GCA GAA GAC GGC ATA CGA GAT TCG ACA AGG TGA CTG GAG TTC AGA CGT GT	16488.7	1.863932898	30.73388444	536500	50.94339623	69.79084006	Standard Desalting	N/A	6nmol	N/A	N/A	N/A	12/14/2021 13:43	
17195044	285828343	354978064	rhAmpSeq™ Index 6 (i7) Primer, 6 nmol	Standard Desalting	i7_IDT-6		0.025	53	CAA GCA GAA GAC GGC ATA CGA GAT CCT TGG AAG TGA CTG GAG TTC AGA CGT GT	16479.7	1.881113619	31.00021821	531600	50.94339623	69.7577081	Standard Desalting	N/A	6nmol	N/A	N/A	N/A	12/14/2021 13:43	
17195044	285828344	354978065	rhAmpSeq™ Index 7 (i7) Primer, 6 nmol	Standard Desalting	i7_IDT-7		0.025	53	CAA GCA GAA GAC GGC ATA CGA GAT ATC ATG CGG TGA CTG GAG TTC AGA CGT GT	16479.7	1.875468867	30.9071943	533200	50.94339623	69.78343422	Standard Desalting	N/A	6nmol	N/A	N/A	N/A	12/14/2021 13:43	
17195044	285828345	354978066	rhAmpSeq™ Index 8 (i7) Primer, 6 nmol	Standard Desalting	i7_IDT-8		0.025	53	CAA GCA GAA GAC GGC ATA CGA GAT TGT TCC GTG TGA CTG GAG TTC AGA CGT GT	16461.7	1.906941266	31.39147597	524400	50.94339623	69.95094119	Standard Desalting	N/A	6nmol	N/A	N/A	N/A	12/14/2021 13:43	
17195044	285828346	354978067	rhAmpSeq™ Index 9 (i7) Primer, 6 nmol	Standard Desalting	i7_IDT-9		0.025	53	CAA GCA GAA GAC GGC ATA CGA GAT ATT AGC CGG TGA CTG GAG TTC AGA CGT GT	16479.7	1.873360809	30.8724541	533800	50.94339623	69.63533178	Standard Desalting	N/A	6nmol	N/A	N/A	N/A	12/14/2021 13:43	
17195044	285828347	354978068	rhAmpSeq™ Index 10 (i7) Primer, 6 nmol	Standard Desalting	i7_IDT-10		0.025	53	CAA GCA GAA GAC GGC ATA CGA GAT CGA TCG ATG TGA CTG GAG TTC AGA CGT GT	16479.7	1.872659176	30.86089139	534000	50.94339623	69.55917854	Standard Desalting	N/A	6nmol	N/A	N/A	N/A	12/14/2021 13:43	
17195044	285828348	354978069	rhAmpSeq™ Index 11 (i7) Primer, 6 nmol	Standard Desalting	i7_IDT-11		0.025	53	CAA GCA GAA GAC GGC ATA CGA GAT GAT CTT GCG TGA CTG GAG TTC AGA CGT GT	16470.7	1.897173212	31.24777651	527100	50.94339623	69.87549244	Standard Desalting	N/A	6nmol	N/A	N/A	N/A	12/14/2021 13:43	
17195044	285828349	354977974	rhAmpSeq™ Index 12 (i7) Primer, 6 nmol	Standard Desalting	i7_IDT-12		0.025	53	CAA GCA GAA GAC GGC ATA CGA GAT AGG ATA GCG TGA CTG GAG TTC AGA CGT GT	16528.8	1.84569952	30.50711148	541800	50.94339623	69.4070305	Standard Desalting	N/A	6nmol	N/A	N/A	N/A	12/14/2021 13:43	
