# Supplementary material for: Targeted NGS-Based Analysis of Pneumocystis jirovecii Reveals Novel Genotypes
Source: J Fungi (Basel). 2022 Aug 17;8(8):863. doi: 10.3390/jof8080863 (PMC9409852; doi:10.3390/jof8080863)
Supplement: Supplementary file 1 [file jof-08-00863-s001.zip › SupplementaryFiles/Supplemental Table 2.pdf]

Supplemental Table 2: full SNP table of *P. jiroveci* genomic SNPs

Ref ID	Ref Pos	Gene Name	Transcript ID	Murina Ortholog	B1	B2	B3	B4	B5	M1	M2	M3	M4	M5	M6	T1	T2	T3	T4	T5	T6	Z1	Z2	Z3	Z4	Z5	Z6	C1	C2	C3	C4	C5	C6	C7	C8	C9	C10	C11	U1	U2
					4 months	20 months	4 months	3 months	8 months	4 months	2 months	5 months	4 months	3 months	5 months	4 months	3 months	2 months	3 months	2 months	2 months	2 months	3 months	3 months	2 months	2 months	3 months	37 years	30 years	49 years	37 years	46 years	83 years	54 years	63 years	79 years	75 years	52 years	32 years	31 years
					Male	Male	Male	Female	Male	Female	Male	Male	Male	Male	Male	Male	Male	Female	Female	Female	Female	Male	Male	Female	Male	Female	Female	Male	Female	Male	Male	Male	Female	Male	Female	Male	Male	Female	Male	Male
					HIV negative	HIV negative	HIV negative	HIV negative	HIV negative	HIV negative	HIV negative	HIV unknown	HIV unknown	HIV positive	HIV unknown	HIV negative	HIV negative	HIV negative	HIV negative	HIV negative	HIV negative	HIV negative	HIV positive	HIV negative	HIV negative	HIV negative	HIV positive	HIV unknown	HIV unknown	HIV unknown	HIV unknown	HIV unknown	HIV unknown	HIV unknown	HIV unknown	HIV unknown	HIV unknown	HIV unknown	HIV positive	HIV positive
NW_017264775.1	588050	T551_00303	XM_018372570.1	GTP binding									A>G																											
NW_017264775.1	588078	T551_00303	XM_018372570.1	GTP binding																													A>C							
NW_017264775.1	588101	T551_00303		GTP binding																															A>G					
NW_017264775.1	588109	T551_00303		GTP binding																														A>T						
NW_017264775.1	588138	T551_00303	XM_018372570.1	GTP binding																														T>C						
NW_017264784.1	126653	T551_02309	XM_018374572.1	GSC-1											- > ins AT					- > ins AN | ins AT									- > ins AT	- > ins AT		- > ins AT	- > ins AT							- > ins AT
NW_017264784.1	126657	T551_02309	XM_018374572.1	GSC-1											TA > del 2					TA > del 2									TA > del 2	TA > del 2		TA > del 2	TA > del 2							TA > del 2
NW_017264785.1	79679							A>C				A>C	A>C			A>C			A>C			A>C									A>C				A>C					
NW_017264785.1	194152	T551_02533	XM_018374796.1	Meu10	C>T					C>T	C>T			C>T									C>T	C>T																
NW_017264785.1	194184	T551_02533	XM_018374796.1	Meu10	G>A					G>A	G>A			G>A									G>A	G>A																
NW_017264786.1	95346	T551_02650	XM_018374913.1	SUMO-targeted ubiquitin-protein ligase E3 Slx8 *C																							C>A													C>A
NW_017264788.1	37245																			G>T																				
NW_017264788.1	37256																			A>T																				
NW_017264788.1	183203	T551_03038	XM_018375301.1	meiotically up-regulated gene 117 protein *C	G>A														G>A																					
NW_017264789.1	101701	T551_03158	XM_018375421.1	DNAJ																												G>A								
NW_017264789.1	195065	T551_03199	XM_018375462.1	Arp9		T>C																																		
NW_017264789.1	195132	T551_03199	XM_018375462.1	Arp9	A>G											A>G	A>G		A>G																					
NW_017264789.1	195710	T551_03199	XM_018375462.1	Arp9																														A>G						
NW_017264776.1	609401	T551_05004										C>T																												
NW_017264776.1	610345	T551_05004			T>C																																			
NW_017264776.1	610355	T551_05004			C>T																																			
NW_017264776.1	610377	T551_05004			C>T																																			
NW_017264776.1	610382	T551_05004			G>A																																			
NW_017264776.1	610873	T551_05004																		T>A																T>A				
NW_017264776.1	610917	T551_05004																		A>G																A>G				
NW_017264776.1	610922	T551_05004																		C>T																C>T				
NW_017264776.1	612454	T551_05005			C>T																											C>T								
NW_017264776.1	612497	T551_05005			TG>CT																											TG>CT								
NW_017264776.1	613009	T551_05005										T>C																												
NW_017264776.1	613023	T551_05005										A>G																												
NW_017264776.1	613025	T551_05005										T>C																												
NW_017264776.1	614174	T551_05005										A>G																												
NW_017264776.1	614213	T551_05005										T>G																												
NW_017264776.1	614228	T551_05005										TG>GT																												
NW_017264776.1	614236	T551_05005			T>C							T																												
NW_017264776.1	614241	T551_05005			G>T							G>T																												
NW_017264776.1	614245	T551_05005			T>C							T>C																												
NW_017264776.1	614278	T551_05005			AA>GG							AA>GG																												
NW_017264776.1	614321	T551_05005			T>C																																			
NW_017264776.1	614686	T551_05005			C>T																															C>T				
NW_017264776.1	614722	T551_05005			A>G							A>G																								A>G				
NW_017264776.1	614744	T551_05005										T>C																								T>C				
NW_017264776.1	614781	T551_05005																																		ACC>TGT				
NW_017264776.1	614883	T551_05005																																		- > ins T				
NW_017264776.1	614884	T551_05005																																		AA>GT				
NW_017264776.1	614981	T551_05005														T>C																								
NW_017264776.1	614989	T551_05005														T>G																								
NW_017264776.1	614998	T551_05005														G>A																								
NW_017264777.1	577940	T551_00896	XM_018373160.1	Four F5 Protein																												A>G								
NW_017264777.1	577947	T551_00896	XM_018373160.1	Four F5 Protein	G>A			G>A	G>A	G>A					G>A		G>A	G>A	G>A	G>A	G>A	G>A	G>A	G>A	G>A	G>A	G>A					G>A	G>A							G>A
NW_017264777.1	577980	T551_00896		Four F5 Protein																												T>C								
NW_017264777.1	577987	T551_00896		Four F5 Protein																					T>C															
NW_017264777.1	577989	T551_00896		Four F5 Protein											T>C																	T>C	T>C							
NW_017264777.1	578000	T551_00896	XM_018373160.1	Four F5 Protein	C>T			C>T							C>T		C>T	C>T	C>T	C>T	C>T	C>T	C>T	C>T	C>T	C>T	C>T					C>T	C>T							C>T
NW_017264781.1	237900	T551_01756	XM_018374019.1	Arc*C																	C>T																			
NW_017264781.1	389537	T551_01830	XM_018374093.1	hsp72-like protein (hsp 70*C)								C>T									C>T				C>T							C>T			C>T					
NW_017264781.1	389571	T551_01830	XM_018374093.1	hsp72-like protein (hsp 70*C)			T>A		T>A		T>A						T>A	T>A				T>A							TC>AG											
NW_017264781.1	389572	T551_01830	XM_018374093.1	hsp72-like protein (hsp 70*C)																		C>G																		

Sample ID Legend	
Sample ID	Origin
B1-B5	Bangladesh
M1-M6	Mali
T1-T6	Thailand
Z1-Z6	Zambia
C1-C11	Canada
U1-U2	United States
