# Supplementary material for: Targeted NGS-Based Analysis of Pneumocystis jirovecii Reveals Novel Genotypes
Source: J Fungi (Basel). 2022 Aug 17;8(8):863. doi: 10.3390/jof8080863 (PMC9409852; doi:10.3390/jof8080863)
Supplement: Supplementary file 1 [file jof-08-00863-s001.zip › SupplementaryFiles/Supplemental Table 3.pdf]

Supplemental Table 3: full SNP table of *P. jiroveci* mitochondrial SNPs

Ref ID	Ref Pos	Gene Name	B1	B2	B3	B4	B5	M1	M2	M3	M4	M5	M6	T1	T2	T3	T4	T5	T6	Z1	Z2	Z3	Z4	Z5	Z6	C1	C2	C3	C4	C5	C6	C7	C8	C9	C10	C11	U1	U2
			4 months	20 months	4 months	3 months	8 months	4 months	2 months	5 months	4 months	3 months	5 months	4 months	3 months	2 months	3 months	2 months	2 months	2 months	3 months	3 months	2 months	2 months	3 months	37 years	30 years	49 years	37 years	46 years	83 years	54 years	63 years	79 years	75 years	52 years	32 years	31 years
			Male	Male	Male	Female	Male	Female	Male	Male	Male	Male	Male	Male	Male	Female	Female	Female	Female	Male	Male	Female	Male	Female	Female	Male	Female	Male	Male	Male	Female	Male	Female	Male	Male	Female	Male	Male
			HIV negative	HIV negative	HIV negative	HIV negative	HIV negative	HIV negative	HIV negative	HIV unknown	HIV unknown	HIV positive	HIV unknown	HIV negative	HIV negative	HIV negative	HIV negative	HIV negative	HIV negative	HIV negative	HIV positive	HIV negative	HIV negative	HIV negative	HIV positive	HIV unknown	HIV unknown	HIV unknown	HIV unknown	HIV unknown	HIV unknown	HIV unknown	HIV unknown	HIV unknown	HIV unknown	HIV unknown	HIV positive	HIV positive
NC_020331.1	17794	cox1													- > ins C	A																						
NC_020331.1	17826	cox1																												A>G			A					
NC_020331.1	17847						C>A					C>A					C>A			C>A	C>A	C>A	C	C>A	C		C>A			C>A			C>A					
NC_020331.1	17850						C>T		C>T			C>T					C>T			C>T	C>T	C>T	C	C>T	C>T		C>T			C>T			C>T					C>T
NC_020331.1	17858						- > ins T					- > ins T					- > ins T			- > ins Tx4				- > ins T	C					- > ins T			- > ins T					
NC_020331.1	17860																			T > del 1	T>G	T																
NC_020331.1	17861																			TTT > del 3	T																	
NC_020331.1	17864						G>T					G>T					G>T							G>T	G					G>T			G>T					
NC_020331.1	17928	atp8										G>T					G>T			G>T	G>T	G>T	G	G>T	G>T		G>T			G>T			G>T					G>T
NC_020331.1	18370	atp6											G>C					G (NC)																				
NC_020331.1	18373	atp6											A>T					A																				
NC_020331.1	18383	atp6					- > ins G								T																							
NC_020331.1	18387	atp6				- > ins T	C								C																							
NC_020331.1	19892	cox3											T>C					T (NC)																				
NC_020331.1	19895	cox3											A>C					A (NC)																				
NC_020331.1	19897	cox3											T>G					T (NC)																				
NC_020331.1	19899	cox3											G>T					G (NC)																				
NC_020331.1	19910	cox3						T															- > ins T															
NC_020331.1	19949	cox3														C>T	C																					
NC_020331.1	20254	atp9														- > ins G	A (NC)																					
NC_020331.1	20259	atp9				- > ins A	T			- > ins A																												
NC_020331.1	20361	atp9			T					T>C																												
NC_020331.1	20676	nad4L													- > ins G								- > ins G				T		T (NC)									
NC_020331.1	20682	nad4L																	- > ins A								T											
NC_020331.1	20688	nad4L			A					- > ins T											A>G, - > ins A																	
NC_020331.1	20783	nad4L				C>T													C>T								C											
NC_020331.1	23537	nad4			T>C	T																																
NC_020331.1	23539	nad4			- > ins T	- > ins G	- > ins T						T	- > ins T	- > ins G						- > ins T						- > ins T		T (NC)									
NC_020331.1	29181	nad6			T		T											- > ins G				T	- > ins G		- > ins G													
NC_020331.1	29185	nad6			T						- > ins G																											
NC_020331.1	29190	nad6																	- > ins T								C											
NC_020331.1	29320	nad6	T									T						T>A		T>A	T (NC)																	
NC_020331.1	30066	nad1								- > ins A		A				- > ins T	- > ins T																					
NC_020331.1	30069	nad1			A						- > ins A												- > ins G															
NC_020331.1	30077	nad1													- > ins T	T																						

Sample ID Legend	
Sample ID	Origin
B1-B5	Bangladesh
M1-M6	Mali
T1-T6	Thailand
Z1-Z6	Zambia
C1-C11	Canada
U1-U2	United States
